# Poor-Law Nursing in England. III. The Sick Wards


**Published:** 1917-06-30

**Authors:** 


### THE MATRONS' AND SISTERS' DEPARTMENT.

### POOR-LAW NURSING IN ENGLAND.

### III.—The Sick-Wards.*

THE case of the "sick-wards" is very different from that of the large infirmaries in the first and second class in Poor Law.

They are completely part of the workhouse, and though usually in a separate wing of the building, they have no separate existence.

The superintendent nurse ranks below the master and matron, and is under their authority in all matters not actually connected with the treatment of the sick. For this she is responsible to the medical officer, who is non-resident in most of the small workhouses. She is often the only trained nurse, with inexperienced assistant nurses to help her. She is called up in the night if a patient gets worse or if there is any sudden emergency in the workhouse, and has to attend the midwifery cases if there is no qualified midwife. Most of the patients are infirm, chronic, and incurable cases, and the lack of variety makes the work very monotonous. The hours are long and the off-duty time uncertain, as the doctor, generally a busy man with a large practice, has no fixed time for visiting the wards. Often he is in a hurry and only sees the most serious cases, leaving the rest, with a few general directions and standing orders, to the superintendent nurse. This may be very good experience; but it is also a great strain on a conscientious woman.

At times it is hard to resist the temptation to get slack about things when there is no one who takes any interest in her work, or troubles about it as long as the patients seem comfortable and make no complaints.

In these small places the superintendent nurse is cut off from friendly intercourse with other hospitals, and is thrown back on the society of the workhouse officials. Gradually she comes under their influence, descends more or less to their level, and her professional standard is lowered.

If she has a strong personality, and stands out against the workhouse influence, she will find herself in an isolated position and will probably, if keen for improvements and reforms, find the workhouse authorities, if not the Guardians, against her. Everything she does will be criticised and the worst possible construction put upon it. The close connection between the workhouse and "sick-wards" makes this an easy matter. Everything required for the wards and patients must be asked for, and is supplied by the master or matron. If a larger supply of clothing is asked for, it is considered extravagant. There is likely to be a difference of opinion over the worn-out garments sent to be exchanged, or over the amount of soiled linen sent daily to the laundry. Necessary repairs in the wards are long delayed, and improvements are often grudgingly granted.

One of the chief difficulties of the superintendent nurse is that her authority with the nursing staff is not paramount. An insubordinate nurse can appeal to the master if she dislikes an order, or cannot get what she wants. The master may support the superintendent nurse; but the fact that he can reverse her decisions does not make for good discipline.

The system of employing assistant nurses in "sick wards" is far from satisfactory. They are often girls of a lower class and without much education; but they must have had some hospital experience. This usually consists of a year in a home for incurables, or some training in a small hospital for special diseases, such as an eye or skin hospital. Some are girls who failed to get on in other hospitals for various reasons, and left after a year or eighteen months' experience. These girls think an assistant nurse's post sounds better than a probationer's, and are also tempted by the higher salary offered. They have but scanty knowledge of their work and a great idea of their own importance. They are not inclined to submit to discipline or to the authority of the superintendent nurse. No one who has not tried it can realise the difficulty of working with assistant nurses, who have all had "previous experience" at different hospitals, are satisfied with their training, and resent any attempt to alter or improve their methods of doing their work. These girls rank as nurses in the Poor-Law service, but are not *trained* nurses in any real sense of the word. They cannot rise to any responsible nursing post, and rarely stay long anywhere, but drift from one workhouse to another, or join some second-rate nursing home.

Owing to the many difficulties with assistant nurses, these small places are now allowed by the L.G.B. to take probationers for three years. But this system is even more indefensible than that of assistant nurses. It is not possible to give a good and sufficient three years' training, or a certificate worth having, from these workhouse sick-wards. The superintendent nurse greatly prefers probationers whom she can teach and train to the assistant nurses; but it is obviously unfair to the probationers to let them take these posts, only to find out, after three years' hard work, that their certificates are of little or no value, and they must begin again if they wish to rise in their profession. The problem of nursing in small workhouses is a difficult one; but it is surely a grave mistake to create a large class of half-trained or badly trained nurses by engaging assistant nurses and allowing probationers to be trained in small unions. They are not eligible for the post of superintendent nurse, nor should the certificate granted to them be

* Previous articles appeared on June 16, p. 211, and June 23, p. 231.

accepted by any board set up for the registration of trained nurses in this country. In large workhouses the attendants on the imbecile and infirm are sometimes called nurses, and are returned as such on official lists. This probably accounts for the very large number of nurses given by the N.P.L.O.A. as being in the Poor-Law Service. But though these attendants may have had some experience, they are not, in the modern sense, nurses at all, and it is a grave injustice to the well-trained Poor-Law nurse to be classed with them.

It has been suggested that probationers should be passed on from the small to the large infirmaries, after the first year, to complete their training. But one of the chief objections to this plan is that they have so much to unlearn. They have acquired the workhouse tone and manner of doing their work and of speaking to the patients. They have little idea of hospital etiquette or of respect for authority, and their influence amongst junior nurses is not good. Matrons are loth to take girls from small workhouses for these reasons, and prefer those with no experience.

The best solution would be to remove all the sick from all the workhouses and nurse them in separated infirmaries and homes for incurables.

These homes should be under a good nurse-matron, who would take girls for a year's preliminary training and then pass them on to the large infirmaries. The post of superintendent nurse should be abolished with the workhouse sick-wards.

The first and second grades of Poor-Law infirmaries should be merged into one, and the third grade should cease to exist. There is little chance of levelling up all Poor-Law nurses to a higher standard until this is done.

All nurses will then have the same opportunities of a thorough and comprehensive training that are now enjoyed by the probationers in the best infirmary training-schools.

